# Ethical Challenges of Bioprinted and Lab-Grown Skin Substitutes in Burn Care

**DOI:** 10.1093/jbcr/irag125

**Published:** 2026-07-22

**Authors:** Joshua Khorsandi, Brian Mansoury, Abu-Bakr Ahmed, Liahm Blank, Michael Harouni, Demitri Franzoni, Joshua MacDavid

**Affiliations:** Department of Plastic and Reconstructive Surgery, Kirk Kerkorian School of Medicine at University of Nevada Las Vegas, Las Vegas, NV 89106, United States; Department of Plastic and Reconstructive Surgery, Kirk Kerkorian School of Medicine at University of Nevada Las Vegas, Las Vegas, NV 89106, United States; Department of Plastic and Reconstructive Surgery, Kirk Kerkorian School of Medicine at University of Nevada Las Vegas, Las Vegas, NV 89106, United States; Department of Plastic and Reconstructive Surgery, Kirk Kerkorian School of Medicine at University of Nevada Las Vegas, Las Vegas, NV 89106, United States; Department of Plastic and Reconstructive Surgery, Kirk Kerkorian School of Medicine at University of Nevada Las Vegas, Las Vegas, NV 89106, United States; Department of Plastic and Reconstructive Surgery, University of Nevada Las Vegas, Las Vegas, NV 89106, United States; Department of Plastic and Reconstructive Surgery, University of Nevada Las Vegas, Las Vegas, NV 89106, United States

**Keywords:** bioprinted skin, lab-grown skin substitutes, tissue engineering ethics, intellectual property

## Abstract

Severe burns continue to impose significant global morbidity and mortality, particularly where surgical and critical-care resources are limited. As survival has improved, attention has shifted toward optimizing reconstruction. Bioprinted and lab-grown skin substitutes—building on advances in tissue engineering, cellular scaffolds, and 3D bioprinting—offer new possibilities for coverage, yet raise ethical, legal, and distributive challenges that remain underexamined in burn care. A narrative review was conducted using PubMed, Embase, Web of Science, and Google Scholar (2010-2025), supplemented by foundational legal and bioethics literature. Sources were selected for conceptual and policy relevance to ownership, consent, commercialization, intellectual property (IP), and equity in regenerative skin technologies. Fifty publications spanning burn surgery, biobanking, organoid governance, IP law, and global health were synthesized inductively. Five major ethical domains emerged: (1) ambiguous ownership and ontological status of hybrid bioprinted tissues; (2) inadequacy of consent processes to address commercialization and downstream uses of patient-derived cells; (3) complex IP environments that may restrict access and clinical innovation; (4) distributive injustice driven by high costs and global resource disparities; and (5) a near absence of empirical ethics research involving burn patients or care teams. Bioprinted and lab-grown skin substitutes could transform burn reconstruction, but current ethical and regulatory frameworks lag behind. Addressing ownership, consent, commercialization, and equity—supported by robust empirical engagement with patients—is essential for responsible integration of these technologies into burn care.

## INTRODUCTION

Severe burns remain a major cause of morbidity and mortality worldwide, particularly among children and adults in low- and middle-income countries where flame and scald injuries are common and intensive care capacity is limited.^1^^,^^2^ Advances in resuscitation, infection control, critical care, and reconstructive surgery have significantly improved survival, shifting the focus toward long-term functional recovery and quality of life. Nevertheless, deep partial-thickness and full-thickness burns still require timely excision and coverage with autologous split-thickness grafts or, when donor sites are insufficient, use of temporary or permanent skin substitutes.

Over the past 3 decades, tissue engineering and regenerative medicine have transformed the landscape of skin replacement. Early acellular dermal matrices and allogeneic or xenogeneic scaffolds have been joined by more complex, bilayer, and cellular constructs that incorporate keratinocytes, fibroblasts, and stem cells to approximate native skin structure and function.^3^^,^^4^ Recent reviews of tissue-engineered skin emphasize the emergence of prevascularized and pigmented grafts, integration of growth factors, and use of advanced fabrication techniques, including 3D printing, to address large defects and minimize hypertrophic scarring.^5^^,^^6^ In parallel, bioprinting technologies that deposit cell-laden bioinks layer by layer are being developed to generate customizable, anatomically conforming skin constructs for burn patients.^7^^,^^8^

Within burn care, these technologies are not purely speculative. Cultured epithelial autografts and dermo-epidermal autologous substitutes have been used clinically in major burns, demonstrating the feasibility of lab-expanded cells and tissue constructs as part of standard burn reconstruction.^9^^,^^10^ Reviews of skin tissue engineering for burns explicitly frame these developments as a crucial “next step” for difficult-to-cover wounds and for reducing donor-site morbidity.^11^

In contrast, ethical analysis within the burn literature has historically focused on themes such as consent amid pain and sedation, futility and end-of-life decision making, triage in mass casualty events, and rehabilitation ethics.^12^^,^^13^ These contributions underscore that burn care is already ethically complex, but they seldom address the distinctive issues raised by bioprinted and lab-grown skin substitutes. Meanwhile, in broader regenerative medicine and tissue engineering, scholars have begun to examine questions regarding ownership of human cells, commercialization of biospecimens, and fair access to advanced therapies.^14–16^

Bioprinted and lab-grown skin technologies are progressing rapidly, yet ethical, legal, and distributive justice analyses within burn care remain comparatively underdeveloped. As a result, fundamental questions surrounding ownership of cellular materials, informed consent for tissue use, commercialization of engineered grafts, and equitable access to novel therapies remain insufficiently examined. This review addresses that gap by integrating conceptual, legal, and empirical scholarship to illuminate the distinctive ethical challenges posed by emerging skin biofabrication technologies and to identify priority areas for clinicians, researchers, and policymakers as these innovations move closer to routine clinical application.

## METHODS

A narrative literature search was conducted using PubMed (https://pubmed.ncbi.nlm.nih.gov; accessed 10 September 2025), Embase, Web of Science, and Google Scholar (https://scholar.google.com; accessed 10 September 2025). Search terms included combinations of “burns,” “skin substitutes,” “tissue engineering,” “bioprinting,” “3D bioprinted skin,” “lab-grown skin,” “ethics,” “ownership,” “consent,” “commercialization,” “intellectual property,” “equity,” and “distributive justice.” Searches were limited to English-language publications from 2010 to 2025 to capture contemporary scientific and ethical developments related to regenerative skin technologies. Earlier foundational publications in tissue ownership, biomedical ethics, and landmark legal cases were also retained when they provided essential conceptual grounding.

Because this review was intended to be interpretive rather than systematic, article selection emphasized ethical, legal, and policy relevance rather than strict methodological hierarchy. Eligible sources included peer-reviewed empirical studies, legal analyses, normative bioethics papers, regulatory documents, technology assessments, and professional guidelines pertaining to tissue engineering or regenerative medicine. Publications focused solely on biomaterial performance or engineering optimization were excluded unless they illustrated translational milestones shaping the ethical landscape.

After screening for relevance and conceptual contribution, the final sample consisted of 44 articles spanning burn surgery, regenerative medicine ethics, biobanking, organoid and induced pluripotent stem cell (iPSC) governance, intellectual property (IP) law, and global health. Thematically relevant content from these sources was synthesized inductively to identify the major ethical issues surrounding bioprinted and lab-grown skin substitutes in burn care.

## RESULTS

### Ownership and ontological status of bioprinted tissues

The literature reveals a persistent ambiguity regarding the ontological and legal status of bioprinted tissues created from patient-derived cells. In conventional burn surgery, excised tissue and discarded graft material are rarely conceptualized as property, and US case law has generally held that individuals do not retain property rights in tissues donated for research once they are removed from the body.^17^^,^^18^ The Moore case, discussed in detail by Narayanan, rejected the plaintiff’s claim of property in his excised spleen but emphasized the importance of robust informed consent and fiduciary duties.^19^  Figure 1 provides an overview of the key ethical domains implicated in the development and clinical use of bioprinted and lab-grown skin substitutes in burn care.

**Figure 1:**
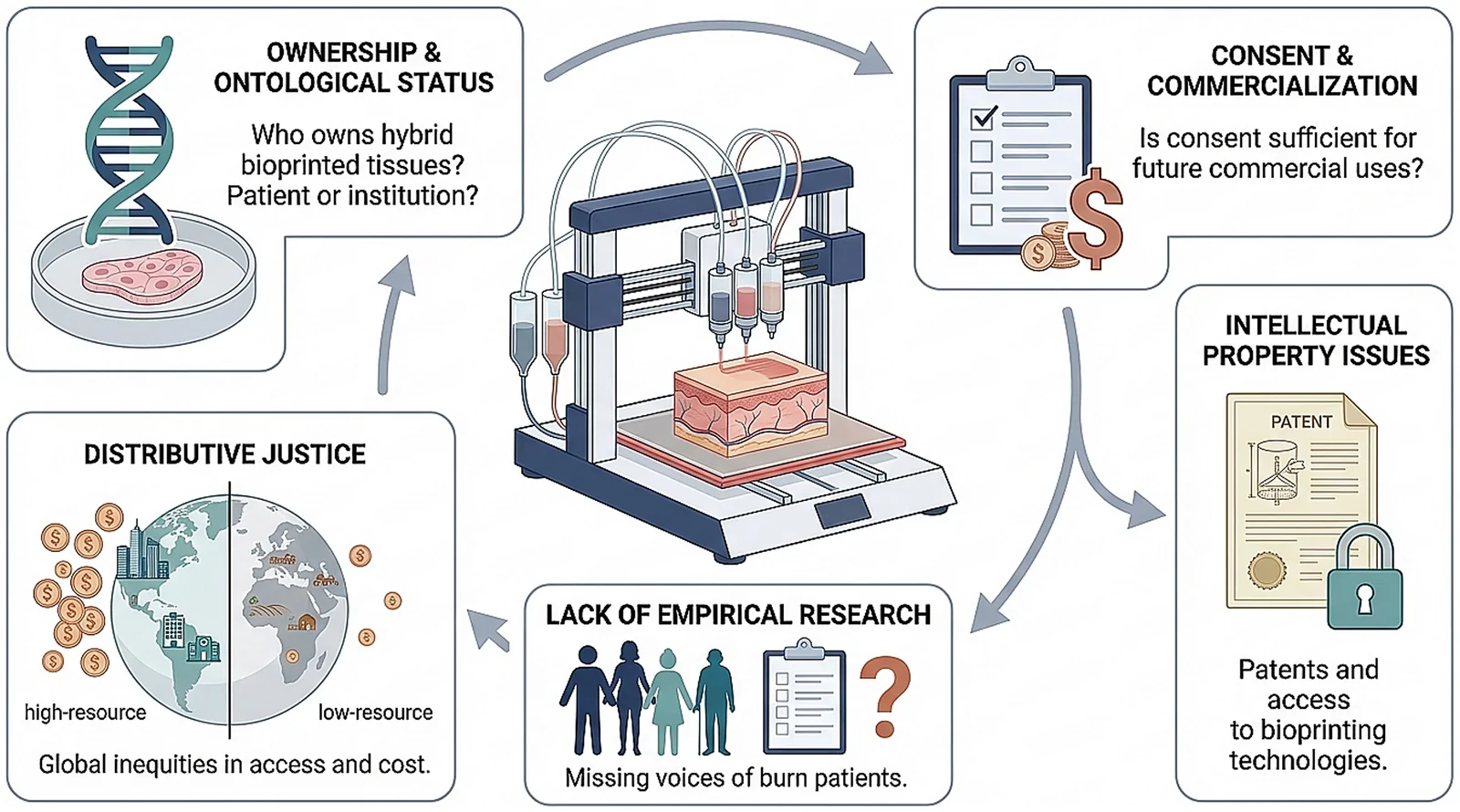
Translational Pathway of Bioprinted and Lab-Grown Skin Substitutes in Burn Care, from Patient Cell Harvesting Through Laboratory Processing, Bioprinting and Fabrication, Clinical Application, and Health System Integration, With Key Ethical Considerations Arising at Each Stage. Image created by authors using FigureLabs. Abbreviation: IP = intellectual property.

Bioprinted skin complicates this landscape by blending human cells with synthetic scaffolds, bioactive molecules, and digital design files derived from patient imaging. Legal commentary on human tissue as property highlights the difficulty of fitting such hybrid constructs into existing categories of body parts, cell lines, and manufactured products.^20^ Some scholars argue that once cells are extensively manipulated and incorporated into novel tissue constructs, they resemble patented biotechnological inventions rather than excised human tissue, potentially shifting ownership toward institutions and companies rather than patients; others caution that ignoring donors’ interests risks repeating the injustices exemplified by the HeLa cell story.^14^

The ontological status of engineered tissues is also problematized in the ethics of organoids and organ-on-chip models, where it has been noted that organoids resist simple classification as cells, tissues, or organs and therefore fall into gaps in current regulatory frameworks.^21^^,^^22^ Bioprinted skin constructs share this liminal character, particularly when integrated with vascular or neural elements, but burn-specific ethical analyses rarely address the conceptual implications of this hybridity. As a result, questions such as whether a bioprinted autologous skin graft remains “part of the patient’s body” or becomes a “product” governed exclusively by device or biologics law remain largely unexamined in the burn literature. Table 1 summarizes the principal ethical domains associated with bioprinted and lab-grown skin substitutes in burn care, including contextual sources, governing frameworks, and key ethical challenges.

**Table 1:** Summary of Ethical Domains, Governing Frameworks, and Key Challenges in Bioprinted and Lab-Grown Skin Substitutes for Burn Care

Ethical domain	Context/data sources	Frameworks/approaches	Key finding(s)
Ownership and ontological status of bioprinted tissues	Legal and bioethics scholarship on excised tissues and organoids	Property law; tissue governance	Bioprinted skin challenges existing legal categories, creating uncertainty over whether patient-derived constructs should be treated as bodily tissue or proprietary biomedical products.^14^
Consent for downstream use and commercialization	Biobanking and regenerative medicine ethics literature	Informed consent models	Standard clinical consent processes inadequately address commercialization and secondary use of patient-derived cells, particularly in acute burn settings.^12^
Commercialization and intellectual property constraints	Patent and policy analyses of bioprinting technologies	Intellectual property law	Proprietary bioprinting platforms and bioink patents may limit interoperability, raise costs, and restrict clinical innovation in burn reconstruction.^23^
Distributive justice and access inequities	Global burn epidemiology and health-economics literature	Distributive justice; global health ethics	High costs of advanced skin substitutes risk concentrating access in high-income settings, potentially widening existing global disparities in burn care.^24^
Global health and regulatory variation	International regulatory analyses of regenerative therapies	Comparative regulatory ethics	Regulatory heterogeneity and hospital-exemption pathways may enable uneven oversight and premature clinical use of bioprinted skin substitutes.^25^

Abbreviations: IP = intellectual property; iPSC = induced pluripotent stem cell.

### Consent and commercialization of biospecimens

The consent literature in biobanking and regenerative medicine repeatedly emphasizes that standard clinical consent forms often fail to address downstream commercialization, broad data sharing, and secondary research uses.^14^^,^^26^ Studies of donors’ attitudes show ongoing concerns about profit-making from donated samples and a desire for transparency and, in some contexts, benefit-sharing arrangements.^27^ These concerns are echoed in discussions of iPSC and organoid research, where donor control and the prospect of commercial applications are central themes.^28^

In burn care, the need for rapid decision making, intense pain, and sedation complicate consent for skin biopsies used to generate cultured autografts or experimental engineered substitutes.^13^^,^^29^ Yet, few empirical studies examine how burn patients understand or value the potential commercialization of their cells when consenting to such procedures. Commentary on tissue banking suggests that participants often do not anticipate commercial uses, particularly when the initial context is framed as clinical care rather than research.^14^^,^^17^ The convergence of time-critical burn surgery and complex, evolving downstream uses of biospecimens raises normative questions about the adequacy of single, broad consent models. Some organoid and iPSC ethics frameworks call for tiered consent options, ongoing communication, and community engagement, especially when samples are drawn from populations who have historically experienced exploitation.^21^^,^^27^ The burn literature has yet to integrate these approaches systematically into consent processes for bioprinted or lab-grown skin substitutes.

### Intellectual property disputes and proprietary bioprinting technologies

Bioprinting literature highlights an increasingly complex IP environment involving patents on bioprinters, bioinks, scaffold materials, and design algorithms, as well as potential IP claims over bioprinted tissues themselves.^23^ Kantaros and colleagues describe how proprietary bioink formulations and closed hardware ecosystems may limit interoperability and create vendor lock-in, thereby shaping which clinical applications are feasible and financially sustainable. Similar concerns appear in analyses of patenting biological materials and genetic tests, where broad patents have, at times, hindered downstream research and clinical access.^19^^,^^30^

In the context of skin substitutes, commercial products already occupy a premium segment of the burn market, with high per-square-centimeter costs and sometimes limited reimbursement.^3^^,^^31^ An analysis of commercialization pathways for advanced skin substitutes stresses that companies often rely on robust IP portfolios to recoup development costs, which may reinforce high pricing and narrow indications.^15^ When bioprinting is added to this environment, additional IP layers—for example, protection over patient-specific digital design files derived from imaging data—raise questions about whether patients should have any control or rights over derivative data and products.^23^

The burn-specific literature rarely addresses these issues explicitly, even as proof-of-concept bioprinted skin grafts for wound healing progress in preclinical and early translational work.^7^ Lessons from other fields suggest that uncritical adoption of proprietary platforms may constrain clinical experimentation, limit comparative effectiveness research, and place clinicians in the position of intermediaries between patients and powerful industrial IP holders.^14^^,^^15^

### Distributive justice concerns associated with cost and access

Distributive justice is a prominent theme in the broader regenerative medicine and gene therapy literature, where the extremely high prices of novel treatments have raised concerns about affordability, priority setting, and global inequity.^15^ Analyses of advanced therapy medicinal products in Europe emphasize that regulatory pathways and health technology assessment mechanisms strongly influence which therapies reach markets and which populations benefit, given finite health care budgets.^25^

Burn care already exhibits stark global disparities: most burn deaths occur in low- and middle-income countries, where access to early excision, skin grafting, and intensive care is limited^2^^,^^32^. Studies of burn centers in resource-constrained settings describe reliance on simplified dressings, delayed excision, and limited access to commercial skin substitutes or cadaveric allograft.^33^^,^^34^ Although low-cost, locally produced allografts or xenografts can partially mitigate these constraints, the current generation of tissue-engineered skin substitutes is largely priced beyond the reach of many systems.^3^

The introduction of bioprinted and lab-grown skin substitutes threatens to widen these gaps if product development is driven primarily by high-income markets and protected through strong IP rights. Commentators on regenerative medicine warn that without intentional strategies for tiered pricing, public–private partnerships, or open-source platforms, advanced therapies will remain concentrated in wealthy health systems.^15^ Within burn care, the literature on ethics of triage and resource allocation during disasters underscores longstanding tensions between maximizing outcomes and ensuring fair access when specialized resources, such as ICU beds or ventilators, are scarce.^35^ However, discussions about how to equitably allocate high-cost, technologically complex skin substitutes or bioprinted grafts have not yet entered mainstream burn ethics discourse.

### Global health considerations

Global health perspectives on burn care emphasize prevention, capacity building, and context-appropriate technologies rather than high-end bioengineering solutions.^2^ Reports from international collaborations call for research that directly addresses the needs of low-resource settings, such as low-cost dressings, improved first aid, and scalable surgical training, rather than importing expensive technologies developed for tertiary centers in high-income countries.

In regenerative medicine more broadly, global regulatory analyses reveal significant heterogeneity in oversight and enforcement, leading to both “stem cell tourism” and variable standards for safety and efficacy.^36^ Similar patterns could emerge for bioprinted and lab-grown skin if unproven products or procedures are marketed directly to patients in jurisdictions with weaker regulatory oversight. Concerns have already been raised about clinics offering unregulated regenerative therapies for wound care and aesthetic indications.^37^

At the same time, some authors suggest that frugal or open-hardware bioprinting approaches might eventually enable local production of skin substitutes in resource-limited settings, provided that regulatory frameworks and technical training are in place.^23^ The current literature, however, offers little concrete guidance on how to align bioprinting innovation with global health priorities in burn care, such as reducing preventable mortality and minimizing long-term disability in settings where even basic resuscitation may be challenging.

### Lack of robust empirical ethics research

Across these themes, a consistent finding is the paucity of empirical ethics studies that specifically examine the perspectives of burn patients, families, or burn care teams on bioprinted and lab-grown skin substitutes. A recent review of ethics in burn care identified topics such as informed consent, futility, and end-of-life decisions, but did not address regenerative technologies or engineered skin products in any depth.^12^ Qualitative work exploring ethical challenges in caring for burn patients focuses on pain management, communication, and team dynamics, again without considering emerging biotechnological interventions.^13^

In parallel, organoid and iPSC ethics researchers have increasingly employed empirical methods—interviews, surveys, and deliberative engagement—to explore how donors and communities perceive the risks and benefits of donating cells for advanced technologies.^14^^,^^27^ Mapping reviews in organoid and organ-on-chip ethics emphasize gaps in normative analysis but at least begin to characterize the empirical landscape.^22^ No comparable body of empirical ethics work exists for bioprinted skin in burn care. This gap limits the ability of ethicists and policymakers to ground conceptual models of ownership, consent, and justice in the lived experience and priorities of those most affected.

## DISCUSSION

This narrative review shows that while technical and translational research on skin substitutes and bioprinted grafts is advancing quickly, ethical, legal, and distributive justice frameworks in burn care lag behind. Many of the issues identified—such as donor control over biospecimens, commercialization of cell-derived products, and inequities in access to expensive therapies—have been extensively discussed in regenerative medicine, biobanking, organoid, and iPSC ethics. Yet the burn community has only begun to engage these debates, and often in a general way rather than with attention to the particularities of burn care. Figure 2 illustrates the translational pathway of bioprinted and lab-grown skin substitutes in burn care and highlights key ethical considerations that arise at each stage, from patient cell harvesting to health system integration.

**Figure 2:**
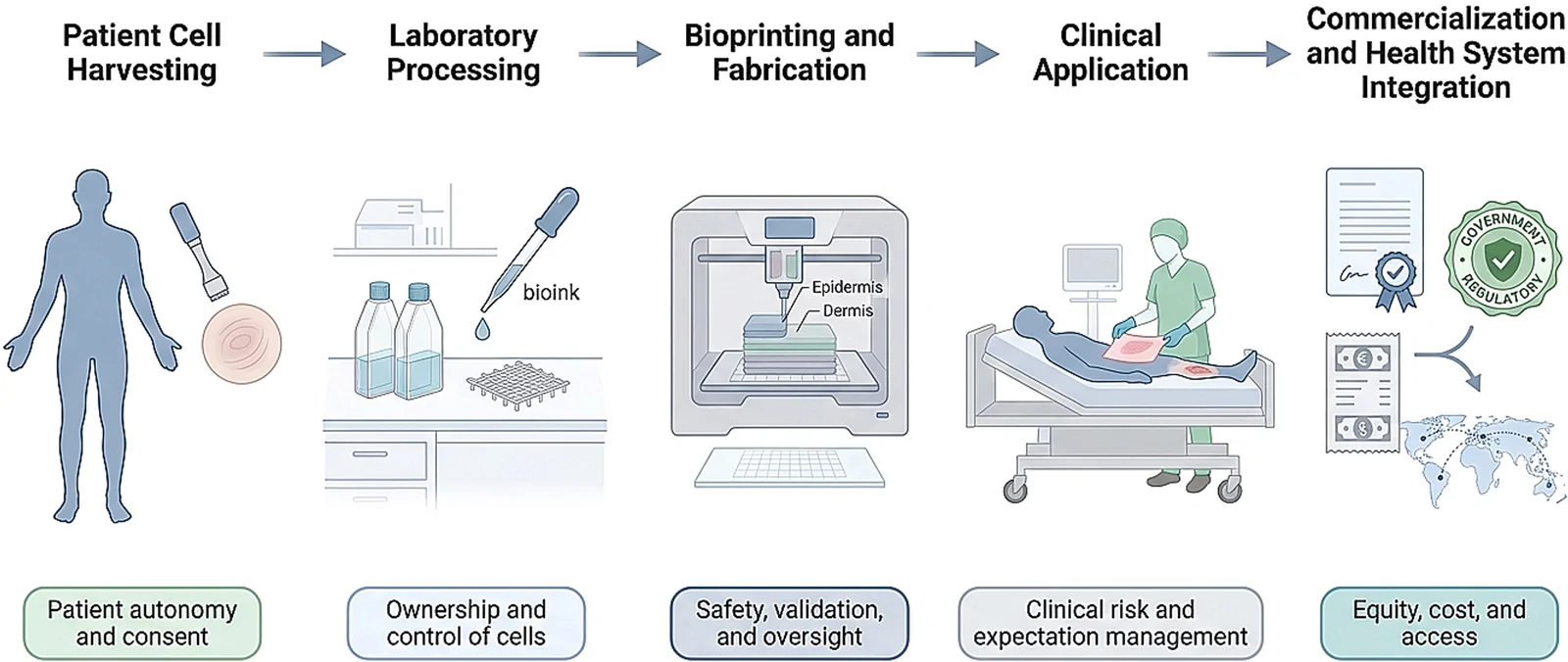
Major Ethical Domains Implicated in the Development and Clinical Use of Bioprinted and Lab-Grown Skin Substitutes for Burn Reconstruction. Image created by authors using FigureLabs.

Comparisons with organoids, gene-edited tissues, iPSCs, and engineered biomaterials are instructive. In organoid ethics, the ambiguous moral and legal status of complex tissue constructs has prompted calls for nuanced governance models that recognize their intermediate status between cells and organs and consider potential future capacities such as neural activity.^21^^,^^22^^,^^38^ Similar concerns about categorization arise for bioprinted skin constructs that incorporate multiple cell types, vascular channels, and perhaps sensory nerve elements, yet little discussion has occurred about whether such constructs should be governed primarily by device, biologic, or tissue regulations in different jurisdictions.

iPSC ethics provides another parallel. Debates about the ethical “advantage” of iPSCs over embryonic stem cells often highlight that iPSCs avoid embryo destruction but introduce new questions about donor privacy, re-identification risks, and possible germline uses.^39^^,^^40^ Meeting reports and empirical studies emphasize the need for consent models that can accommodate a wide range of potential downstream applications, including commercialization, while still respecting participant autonomy.^14^^,^^27^^,^^28^ Bioprinted skin for burn care will likely raise analogous issues as patient cells are reprogrammed, expanded, and incorporated into constructs that may eventually be used beyond the original clinical context, for example, for drug testing platforms or cosmetic applications.

Gene-editing and regenerative medicine ethics more generally underscore that distributive justice cannot be an afterthought. High-priced gene therapies have prompted intense debate over value-based pricing, outcome-based reimbursement, and the risk that life-saving treatments will remain accessible only to a small segment of patients in wealthy health systems.^15^ These debates are especially relevant for burn care, where most global mortality occurs in settings with limited resources, and where failing to address equity in the early phases of technology development may entrench a 2-tier system in which bioprinted skin becomes an option only for patients in well-resourced centers.

At the regulatory level, differences between United States, European, and other international frameworks complicate ethical questions of ownership, consent, and commercialization. In Europe, advanced therapy medicinal products, including tissue-engineered products, are regulated under Regulation (EC) No. 1394/2007, which introduces specialized evaluation pathways but also includes “hospital exemption” provisions that allow certain bespoke therapies to be used without centralized authorization.^25^^,^^41^ In the United States, HCT/P regulations under 21 CFR Part 1271 distinguish between minimally manipulated products regulated primarily for communicable disease control and more substantially manipulated products that are treated as biologics or devices requiring premarket approval.^42^

Bioprinted and lab-grown skin substitutes for burns are likely to fall into the more stringently regulated categories, but hospital-based manufacturing models and compassionate use pathways could blur these boundaries. Regulatory analyses warn that exemptions, if not carefully managed, may be exploited by clinics offering unproven regenerative therapies, raising concerns about patient safety and trust.^36^ For burn care, this suggests a need for clear institutional policies on how bioprinted grafts are produced, validated, and offered, including mechanisms for independent ethics and regulatory oversight.

Within this complex environment, conceptual recommendations emerge for clinicians, institutional review boards (IRBs), researchers, and policymakers. Clinicians who counsel burn patients about participation in trials or clinical use of bioprinted or lab-grown skin should ensure that consent processes explicitly address potential downstream uses of donated tissue, possibilities of commercialization, and uncertainties regarding long-term outcomes. Given the acute setting and the impact of pain and sedation on decision making, staged consent models—where initial agreement to urgent clinical procedures is followed by more detailed discussions about research and commercialization when the patient is more stable—may be ethically preferable.

IRBs and research ethics committees should scrutinize protocols involving bioprinted skin for clarity in ownership and benefit-sharing arrangements and for plans to communicate evolving uses of cell lines and tissue constructs to participants. Drawing on organoid and biobank governance models, IRBs could encourage tiered consent options and community engagement, particularly in settings where burn patients come from marginalized or historically disadvantaged populations.^14^^,^^21^

Researchers and technology developers should consider alternative IP and data-governance models that balance incentives for innovation with equitable access. Options might include non-exclusive licensing for certain applications, openness around bioink formulations that do not rely on patient-specific cells, or collaborative frameworks that ensure public health systems retain leverage in pricing negotiations. Experiences from software, open-source hardware, and global vaccine initiatives suggest that hybrid models can support both innovation and access when designed intentionally.^15^^,^^23^

Policymakers and professional societies in burn care can play a complementary role by developing guidelines that articulate core ethical principles for the clinical introduction of bioprinted and lab-grown skin, including commitments to transparency regarding conflicts of interest and industry partnerships, expectations for post-marketing surveillance of safety and effectiveness, and recommendations for prioritizing equitable access in reimbursement and allocation policies. In the longer term, integration of patient and survivor perspectives into these efforts will be essential to ensure that ethical frameworks reflect the values and priorities of those most affected.

Crucially, burn care presents unique ethical tensions that distinguish it from other regenerative medicine domains. Decisions about using experimental or high-risk reconstructive options often occur in contexts of pain, disfigurement, and psychosocial vulnerability, where patients may be desperate for any intervention that promises improved function or appearance.^29^^,^^43^ The stakes of graft failure can be immediate and life-threatening. These features heighten the risk of therapeutic misconception and underscore the responsibility of clinicians and researchers to avoid overpromising and to manage expectations honestly when introducing novel technologies such as bioprinted skin. Table 2 summarizes the 5 major ethical domains relevant to bioprinted and lab-grown skin substitutes in burn care, outlining each domain’s core ethical concern, implications for burn practice, and key gaps or future priorities for research and policy.

**Table 2:** Ethical Challenges and Future Directions in Bioprinted Skin for Burn Reconstruction

Ethical domain	Core ethical concern	Implications for burn care	Key gap or future priority
Ownership and moral status of engineered skin	Unclear classification of bioprinted skin as tissue, product, or hybrid entity	Ambiguity complicates patient rights, institutional control, and commercialization pathways for autologous bioprinted grafts	Development of burn-specific guidance on ownership and governance of hybrid tissue constructs.^14^
Informed consent under clinical urgency	Acute pain, sedation, and time pressure limit meaningful consent for downstream tissue use	Burn patients may unknowingly consent to commercialization or secondary research uses of their cells	Adoption of staged or tiered consent models tailored to acute burn settings.^12^
Commercialization and intellectual property	Proprietary platforms and bioink patents shape access and pricing	Vendor lock-in and high costs may constrain clinical adoption and comparative research	Exploration of non-exclusive licensing and public–private access models.^23^
Equity and distributive justice	Advanced skin substitutes are costly and resource-intensive	Risk of widening disparities between high-income and low-resource burn care settings	Integration of equity considerations early in technology development and reimbursement policy.^44^
Global health and regulatory oversight	Regulatory heterogeneity and hospital exemptions vary across regions	Uneven oversight may enable premature or unsafe clinical use of bioprinted skin	Harmonization of regulatory standards and safeguards against unproven therapies.^25^

Abbreviation: IP = intellectual property.

### Practical recommendations

The ethical challenges identified in this review are not solely theoretical, and burn clinicians, researchers, IRBs, and professional societies can take deliberate steps now to ensure responsible adoption of these technologies. Burn units should replace single broad consent forms with a staged protocol in which initial consent at biopsy covers immediate clinical use only, with a dedicated follow-up conversation conducted once pain and sedation have resolved addressing research enrollment, tissue banking, and potential commercialization. Clinicians introducing bioprinted skin in trial contexts should also use structured communication that clearly distinguishes experimental from proven therapies, given the heightened risk of therapeutic misconception in this population.

IRBs should apply an explicit checklist to protocols involving bioprinted skin, confirming that consent documents distinguish clinical from research biopsy, disclose commercialization, specify downstream uses of cell lines and digital design files, and address re-contact provisions if anticipated uses change materially. Researchers should engage IP and technology transfer offices early to negotiate non-exclusive licensing for clinical applications and data governance plans specifying ownership of patient-derived digital design files.

The American Burn Association is well positioned to issue a consensus statement covering consent standards, industry disclosure requirements, and equitable access expectations. Priority empirical studies should include qualitative interviews with burn survivors regarding commercialization of their cells and surveys of burn care teams on IP and consent obligations.

### Limitations

This review has several limitations inherent to its narrative design. The literature search, while broad and multi-disciplinary, was not exhaustive, and the absence of formal systematic or scoping methods means that some relevant publications may have been missed. The inclusion of legal analyses, policy documents, and conceptual bioethics papers alongside empirical studies introduces heterogeneity that complicates direct comparison of findings. Publication bias is also likely, as ethical concerns may be under-reported in clinical and engineering journals that prioritize technical outcomes, and legal or ethical critiques may not appear in biomedical databases at all.

Furthermore, the field of bioprinted and lab-grown skin substitutes is rapidly evolving, and some of the technical and regulatory developments discussed here may change within a short time frame. Empirical research on patient, family, and clinician perspectives is particularly sparse, limiting the ability to ground normative analysis in lived experience. Finally, although this review draws on analogous debates from organoids, iPSCs, and other regenerative technologies, not all conclusions can be directly transposed to burn care, where clinical urgency and resource constraints may require different ethical balancing.

## CONCLUSIONS

Bioprinted and lab-grown skin substitutes represent a promising, potentially transformative frontier in burn care, expanding coverage options, reducing donor-site morbidity, and improving functional and cosmetic outcomes, yet ethical and regulatory frameworks have not kept pace with these advances. Unresolved concerns remain regarding ownership and the ontological status of bioprinted tissues, the adequacy of consent for commercialization and long-term use of patient-derived cells, the influence of proprietary platforms and IP regimes, and the possibility that such technologies will deepen global inequities in burn care. To ensure these innovations advance not only technical success but also justice, stakeholders must develop robust ethical guidance that includes transparent, context-sensitive consent processes; fair ownership and benefit-sharing models; and pricing, reimbursement, and allocation strategies that promote equitable access. Expanding empirical ethics research centered on burn patients’ experiences will be essential so that bioprinted and tissue-engineered skin substitutes can be integrated into care in ways that respect autonomy and foster equity. The practical recommendations offered here, including staged consent protocols, IRB checklists, IP negotiation strategies, and professional society guidance, are intended to equip burn clinicians and institutions to begin addressing these challenges proactively rather than reactively once these technologies are already in widespread use.

## Notes

Author contributions: Joshua Khorsandi (Writing—original draft [lead]), Brian Mansoury (Methodology [equal]), Abu-Bakr Ahmed (Formal analysis [lead], Investigation [lead]), Liahm Blank (Conceptualization [lead], Data curation [lead]), Michael Harouni (Data curation [equal]), Demitri Franzoni (Supervision [lead]), Joshua MacDavid (Supervision [equal]).

Funding: This research received no external funding.
